# Supplementary material for: Design of a gold clustering site in an engineered apo-ferritin cage
Source: Commun Chem. 2022 Mar 21;5:39. doi: 10.1038/s42004-022-00651-1 (PMC9814837; doi:10.1038/s42004-022-00651-1)
Supplement: Supplementary file 1 — Supplementary infomation [file 42004_2022_651_MOESM1_ESM.pdf]

Design of a Gold Clustering Site in an Engineered Apo-ferritin Cage

Chenlin Lu[1], Basudev Maity[2], Xue Peng[1], Nozomi Ito[2], Satoshi Abe[2], Xiang Sheng[3], Takafumi Ueno[2] and Diannan Lu[1*]*

1. Department of Chemical Engineering, Tsinghua University, Beijing 100-084, China

2. School of Life Science and Technology, Tokyo Institute of Technology, Yokohama 226-8501, Japan

3. Tianjin Institute of Industrial Biotechnology, Chinese Academy of Sciences, and, National Technology Innovation Center of Synthetic Biology, Tianjin, 300308, China

Corresponding author: ludiannan@tsinghua.edu.cn (D. Lu); tueno@bio.titech.ac.jp (T. Ueno).

CONTENTS

I. Supplementary Note 1

Au binding in apo-R168C/L169C-rHLFr and apo-R161C/L165C-rHLFr.

Figure S1. Screening of Au binding in Cys mutants of ferritin.

II. Supplementary figures

Figure S2. Close view of the four Au binding sites on apo-R168H/L169C-rHLFr Au composites with different Au precursor concentrations.

Figure S3 The anomalous difference Fourier density maps of Au(200 equiv.)-apo-R168H/L169C-rHLFr at 1.15 Å and 1.00 Å.

Figure S4. Three electron density maps at the 4-fold site of apo-R168H/L169C-rHLFr and its Au composites with different Au precursor concentrations.

Figure S5. Clustering at the 3-fold site of apo-R168H/L169C-rHLFr and its Au composites with different Au precursor concentrations.

Figure S6. XPS spectra of apo-R168H/L169C-rHLFr Au composites with different precursor concentrations.

Figure S7. Three possible conformations when the cluster contains four Au ions and corresponding population obtained from Boltzmann distribution based on their Gibbs free energy, respectively.

Figure S8. UV–visible absorption spectrum of apo-R168H/L169C-rHLFr and its Au composites with different Au precursor concentrations measured at day 1, day 3 and day 5.

Figure S9. Excitation Emission Matrix (EEM) spectroscopy of apo-R168H/L169C-rHLFr and its Au composites with different Au precursor concentrations.

III. Supplementary tables

Table S1. Summary of the crystal parameters and refinement statistic parameters for apo-R168H/L169C-rHLFr and Au composites at different equiv.

Table S2. Quantitative analyses (ICP/BCA) of Au atoms per ferritin cage in apo-R168H/L169C-rHLFr Au composites with different precursor concentrations.

Table S3-6. The B-Factors/occupancies of Au atoms and bond distances of Au atoms with adjacent amino acids of Au composites at different equiv.

Table S7. Representative Au–Au, Au-S, Au-N bond distances observed in previous reports.

IV. Supplementary references

I. Supplementary Note 1

Au binding in apo-R168C/L169C-rHLFr and apo-R161C/L165C-rHLFr.

Previously,[1] Cd binding was observed at the 4-fold channel of the apo-R168C/L169C-rHLFr and apo-R161C/L165C-rHLFr where eight cystine acted as coordination residues. In the preliminary Experiments, these two mutants were applied to accumulate Au(I) ions to explore the possibility of Au clustering formation. The Au composites were prepared as described in the Method section, where the Au precursor is 200 equiv. We crystalized the Au(200 equiv.)-apo-R168C/L169C-rHLFr/Au(200 equiv.)-apo-L161C/L166C-rHLFr and solved their crystal structures. The structures are shown in Figure S1, in Au(200 equiv.)-apo-L161C/L166C-rHLFr eight Au ions in total were bound with a similar structure as in the Cd case. Two layers of binding were formed, and in each layer, four Au atoms plus four cystine form a planar structure. In Au(200 equiv.)-apo-R168C/L169C-rHLFr, sixteen Au atoms were observed to bind to the eight cysteines forming a loop structure.

The above preliminary screening experiments demonstrate the potential of the 4-fold channel of apo-ferritin as a site for constructing new-to-nature Au clustering. Another important insight is that the side chains of cysteine are relatively short, making the interaction between Au atoms bound on different monomers rather difficult. In addition, the binding mode is relatively single, i.e., one cysteine bind to two Au atoms acting as a bridge. Therefore, to create a new-to-nature Au clustering site at the 4-fold channel, new coordination residues with longer sidechain and larger rotamer space are needed to build the interaction between Au atoms from different monomers. These screening experiments lead to design of the mutant R168H/L169C mutant which is

described in this paper. In our design, four L169Cs were expected as the main fixing residues and four R168Hs as auxiliary coordinating residues.

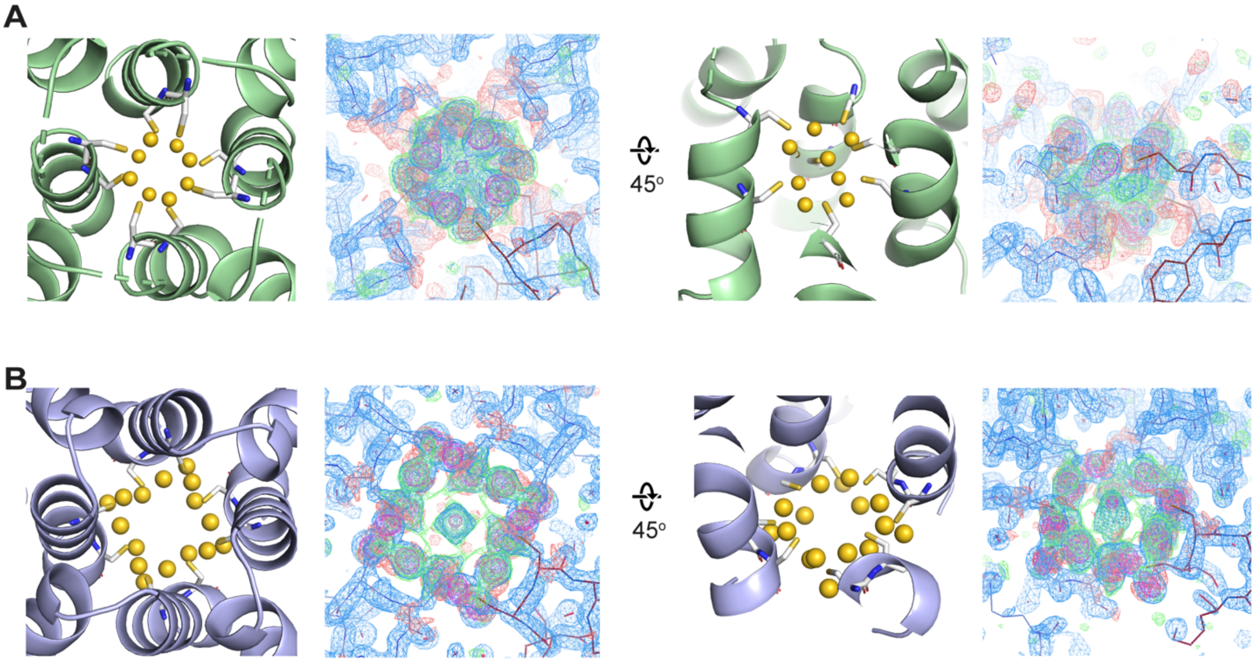

Figure S1. Screening of Au binding in Cys mutants of ferritin. (A)The structure of the Au binding at the 4-fold axis channel of Au(200 equiv.)-apo-L161C/L165C-rHLFr and corresponding electron density maps. (B) The structure of the Au binding at the 4-fold axis channel of Au(200 equiv.)-apo-R168C/L169C-rHLFr and corresponding electron density maps. The Au atoms are shown as yellow spheres. 2Fo-Fc electron density maps at 1σ are shown in the blue, Fo-Fc electron density maps at 3σ are shown in gree(+)/red(-), and anomalous difference Fourier density maps at 4σ are shown in magenta. The major binding positions of Au ions were determined by anomalous electron density maps.

II. Supplementary figures

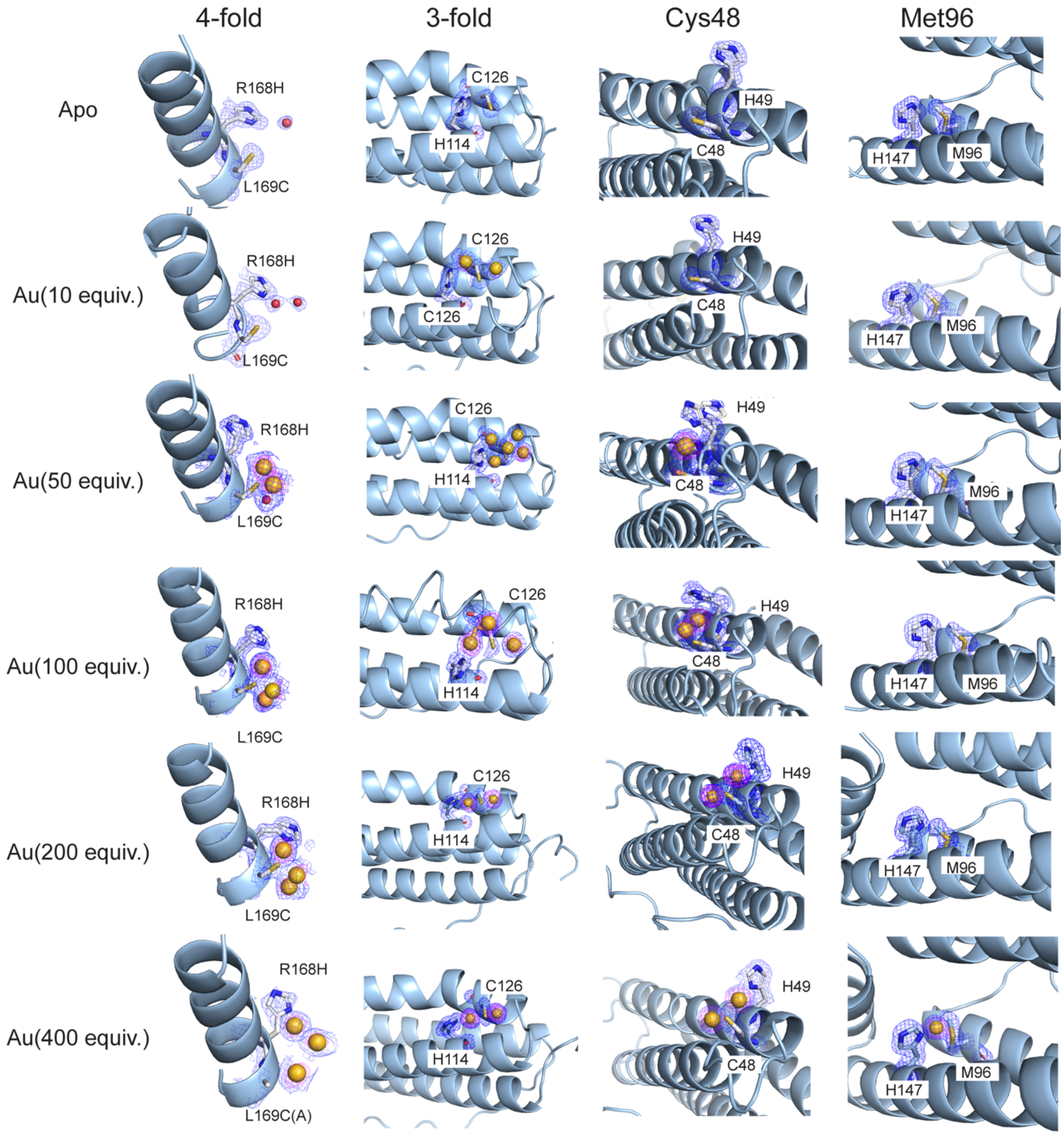

Figure S2. Close view of the four Au binding sites on apo-R168H/L169C-rHLFr Au composites with different Au precursor concentrations. The selected 2Fo-Fc maps at 1σ and anomalous difference Fourier density maps at 4σ are shown in blue and magenta, respectively.

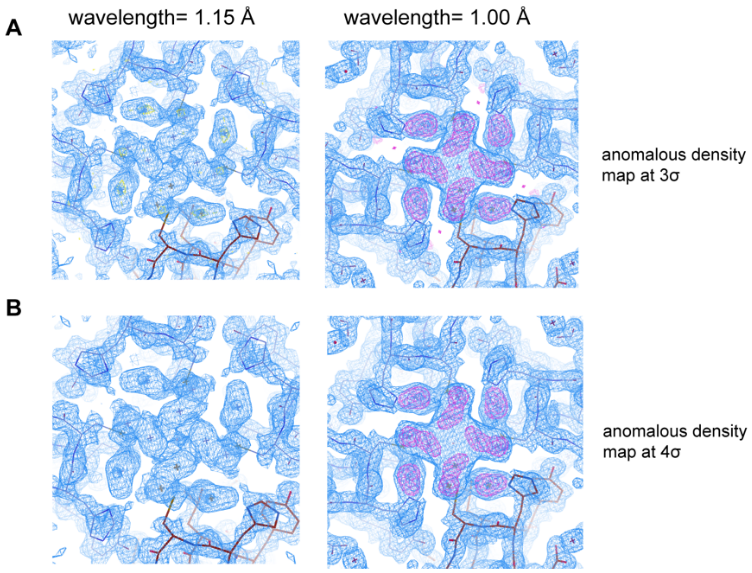

Figure S3. Assignment of Au ions at the 4-fold channel based on anomalous scattering difference at two different wavelengths. (A) The anomalous difference Fourier density maps of Au(200 equiv.)-apo-R168H/L169C-rHLFr at 3σ at 1.15 Å and 1.00 Å wavelength. (B) The anomalous difference Fourier density maps of Au(200 equiv.)-apo-R168H/L169C-rHLFr at 4σ at 1.15 Å and 1.00 Å wavelength. 2Fo-Fc electron density maps at 1σ are shown in blue, and the anomalous density map at 1.15 Å and 1.00 Å were shown in yellow and magenta, respectively.

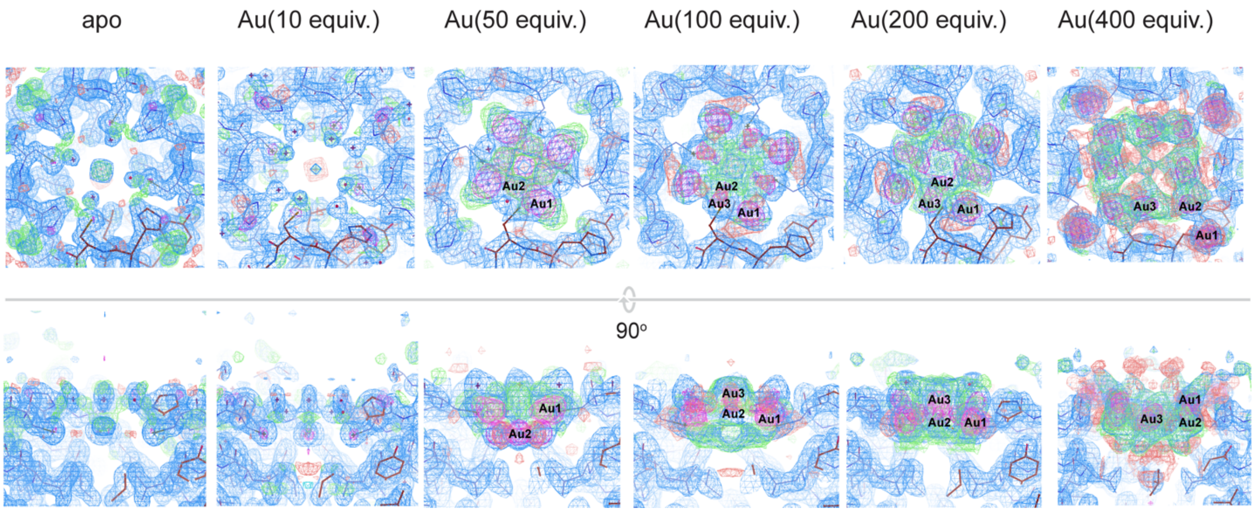

Figure S4. The electron density maps at the 4-fold site of apo-R168H/L169C-rHLFr and its Au composites with different Au precursor concentrations. 2Fo-Fc electron density maps at 1σ are shown in the blue, Fo-Fc electron density maps at 3σ are shown in gree(+)/red(-), and anomalous difference Fourier density maps at 4σ are shown in magenta. Based on anomalous density at 4σ, we assigned the major Au binding positions and occupancies were adjusted considering surrounding B-factors and Fo-Fc difference maps.

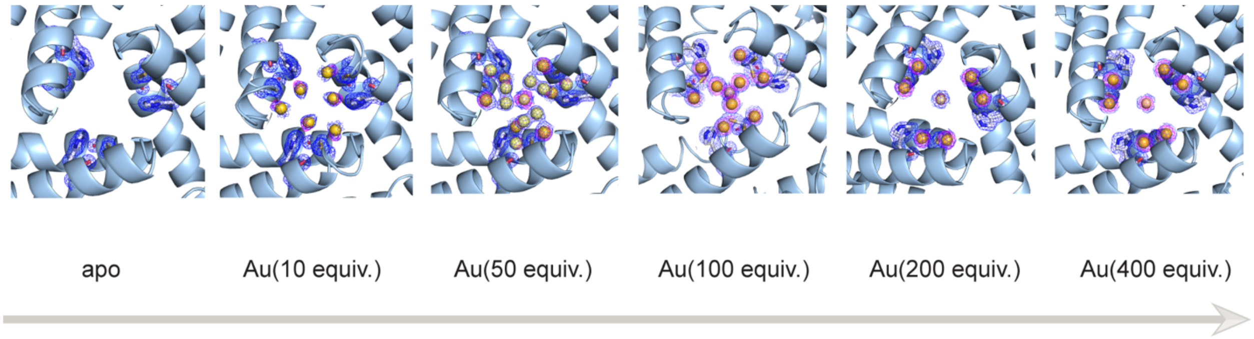

Figure S5. Clustering at the 3-fold site of apo-R168H/L169C-rHLFr and its Au composites with different Au precursor concentrations. The Au atoms are shown as yellow spheres and those with occupancy less than 0.2 were shown in light yellow. 2Fo-Fc electron density maps at 1σ are shown in the blue, and anomalous difference Fourier density maps at 4σ are shown in magenta. The binding positions of Au ions were determined by anomalous electron density maps at 4σ.

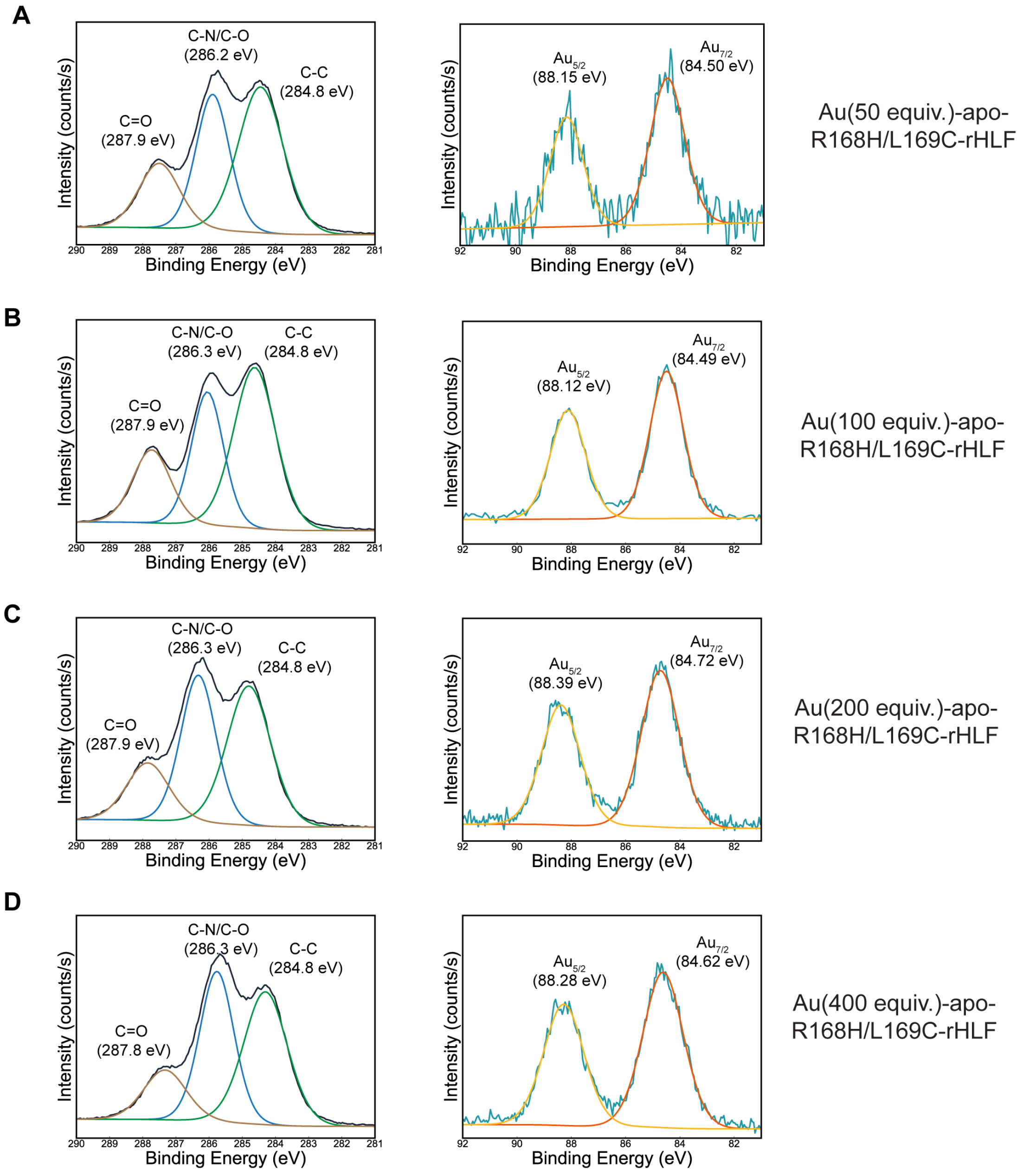

Figure S6. XPS spectra of apo-R168H/L169C-rHLFr Au composites with different precursor concentration. Au(50 equiv.)-apo-R168H/L169C-rHLF (A); Au(50 equiv.)-apo-R168H/L169C-rHLF (B); Au(100 equiv.)-apo-R168H/L169C-rHLF (C); Au(200 equiv.)-apo-R168H/L169C-rHLF (D); Au(400 equiv.)-apo-R168H/L169C-rHLF (E).

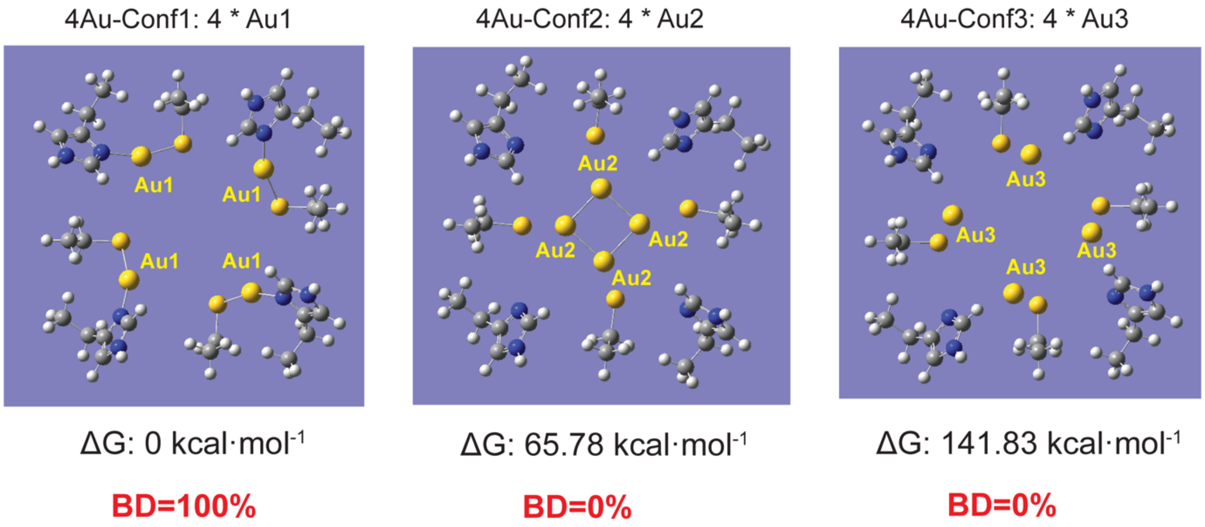

Figure S7. Three possible conformations when the cluster contains four Au ions and corresponding population obtained from Boltzmann distribution based on their Gibbs free energy.

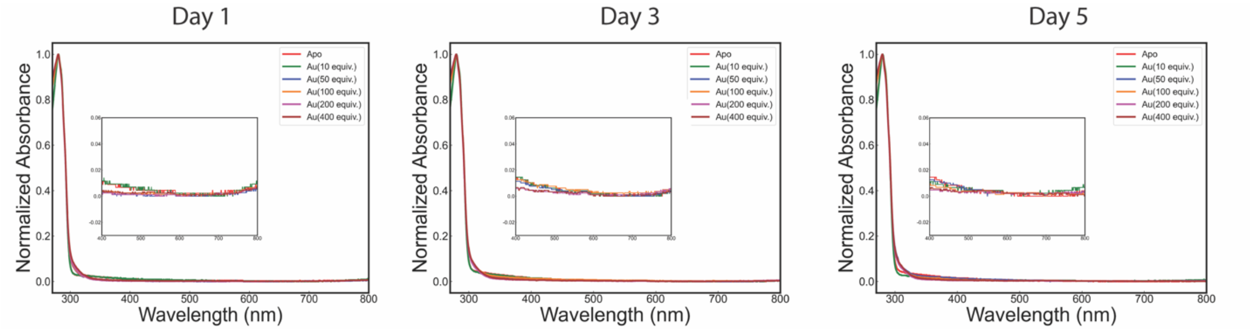

Figure S8. UV–visible absorption spectrum of apo-R168H/L169C-rHLFr and its Au composites with different Au precursor concentrations measured at day 1, day 3 and day 5.

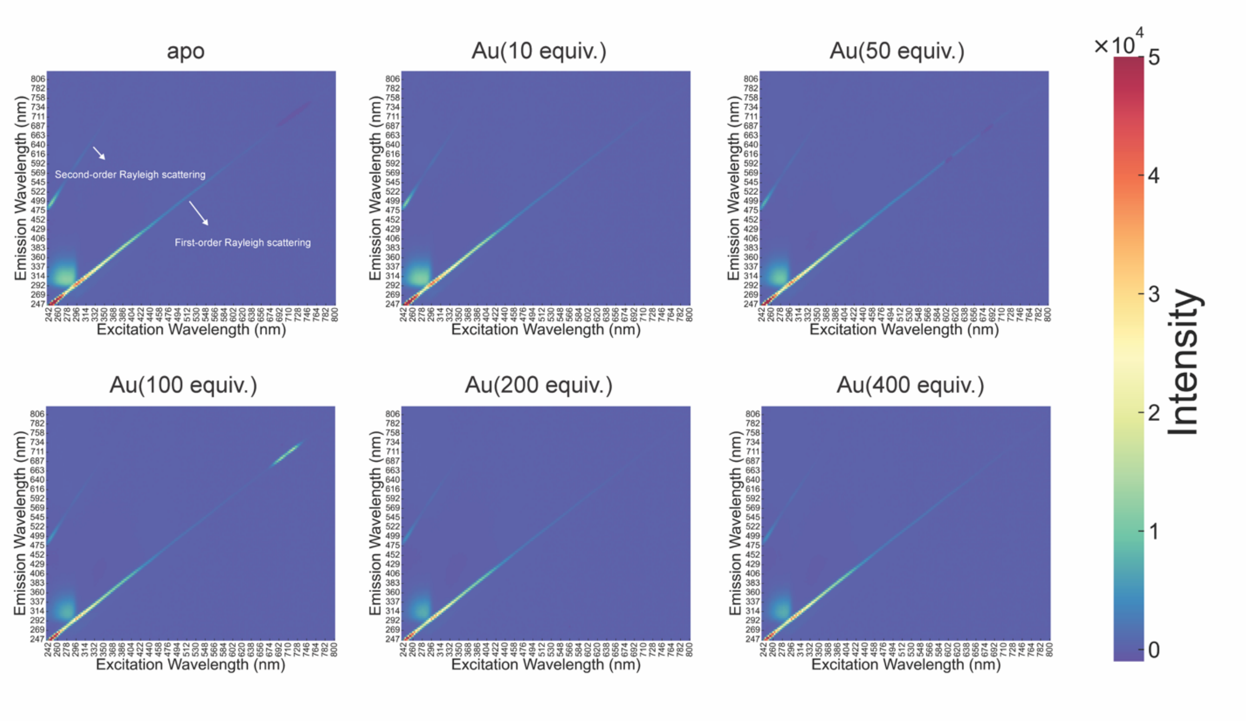

Figure S9. Excitation Emission Matrix (EEM) spectroscopy of apo-R168H/L169C-rHLFr and its Au composites with different Au precursor concentrations.

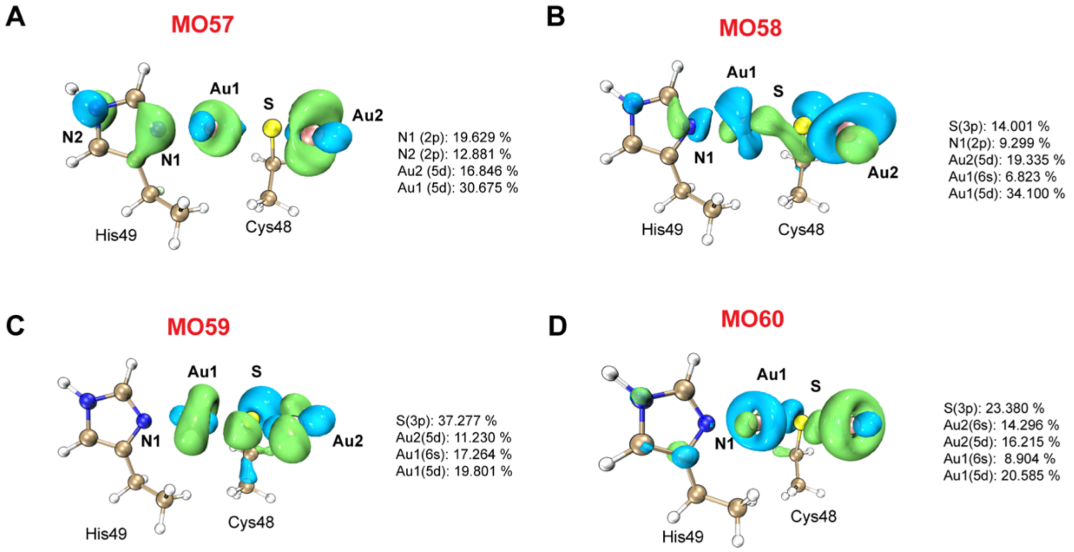

Figure S10. The molecular orbital (MO) that encompasses the Cys48, His49 and coordinated Au atoms with annotation of their major orbital composition contributions (Au(200 equiv.)-apo-R168H/L169C-rHLF).

III. Supplementary tables

Table S1. Summary of the crystal parameters and refinement statistic parameters for apo-R168H/L169C-rHLFr, its Au composites at different equiv, and Au(200 equiv.)-apo-R168C/L169C-rHLFr.

	apo-R168H/L169C-rHLFr	Au(10 equiv.)-apo-R168H/L169C-rHLFr	Au(50 equiv.)-apo-R168H/L169C-rHLFr	Au(100 equiv.)-apo-R168H/L169C-rHLFr	Au(200 equiv.)-apo-R168H/L169C-rHLFr	Au(400 equiv.)-apo-R168H/L169C-rHLFr	Au(200 equiv.)-apo-R168C/L169C-rHLFr
**Data collection statistics**							
Space group	F432	F432	F432	F432	F432	F432	F432
Crystal cell							
a = b = c (Å)	182.64	182.13	180.73	180.21	182.59	180.89	180.79
α = β = γ (º)	90.00	90.00	90.00	90.00	90.00	90.00	90.00
Resolution range (Å)	37.31-1.50	12.88-1.90	12.95–1.90	12.91–1.90	45.69–1.85	12.96–1.90	12.95–1.50
Completeness (%)	99.7	99.7	99.7	99.7	100.0	99.7	99.7
Rmeas	0.039	0.040	0.090	0.096	0.099	0.264	0.082
I/σ	22.7	50.6	25.1	21.5	9.1	43.8	24.2
Facility	SPING8 BL45XU	Rigaku Synergy	Rigaku Synergy	Rigaku Synergy	SPING8 BL45XU	Rigaku Synergy	Rigaku Synergy
**Refinement statistics**							
Resolution (Å)	1.5	1.90	1.9	1.9	1.85	1.9	1.50
Reflection used	42065	20892	20430	20252	22847	20480	401619
R-factor (%)	16.8	18.1	16.8	17.1	16.6	18.3	15.2
Free R-factor (%)	18.8	21.6	19.3	20.9	19.7	21.7	16.7
RMSD from ideal							
Bond length (Å)	0.0164	0.0410	0.0145	0.0119	0.0125	0.0098	0.0115
Angle (°)	2.065	1.739	1.946	1.726	1.764	1.678	1.946
Ramachandran (%)							
Favored	99	99	98	98	98	98	98
Allowed	1	1	2	2	2	2	2

Table S2 Quantitative analysis (ICP/BCA) of Au atoms per ferritin cage in apo-R168H/L169C-rHLFr Au composites with different precursor concentrations.

Sample	ICP-MS/BCA
Au(10 equiv.)-apo-R168H/L169C-rHLF	4±2
Au(50 equiv.)-apo-R168H/L169C-rHLF	46±10
Au(100 equiv.)-apo-R168H/L169C-rHLF	87±8
Au(200 equiv.)-apo-R168H/L169C-rHLF	158±25
Au(400 equiv.)-apo-R168H/L169C-rHLF	203±38
Au(200 equiv.)-apo-R168H/L169C-rHLF (after 5 days' dialysis under 4 °C)	139
Au(200 equiv.)-apo-R168H/L169C-rHLF (after 5 days' dialysis under 20 °C)	137

Table S3 B-Factors (B.F.) and occupancies (Occu.) of metal atoms in Au(50 equiv.)-apo-R168H/L169C-rHLFr

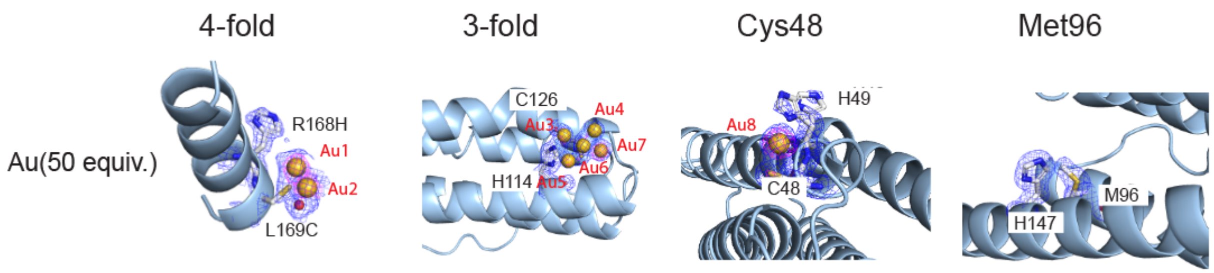

Atom	Au1	Au2	Au3	Au4	Au5	Au6	Au7	Au8
Occu.	0.40	0.30	0.20	0.15	0.15	0.15	0.30	0.50
B.F.(Å^3)	40.86	28.92	32.42	39.08	35.92	33.19	27.34	20.65

Table S4 Bond distances of Au atoms with adjacent amino acids in Au(50 equiv.)-apo-R168H/L169C-rHLFr

Bond	Bond distance (Å)	Bond	Bond distance (Å)
Au1-S^γ (L169C)	1.84	Au5- S^γ (C126)	2.12
Au2-S^γ (L169C)	2.60	Au6- S^γ (C126)	3.42
Au3- N^ε (H114)	1.85	Au7- S^γ (C126)	2.57
Au3- O^ε (E130)	1.80	Au8- S^γ (C48)	2.44
Au4- S^γ (C126)	4.21	Au8- N^ε (H49)	2.11
Au5- N^ε (H114)	2.22		

Table S5 B-Factors (B.F.) and occupancies (Occu.) of Au atoms in Au(100 equiv.)-apo-R168H/L169C-rHLFr

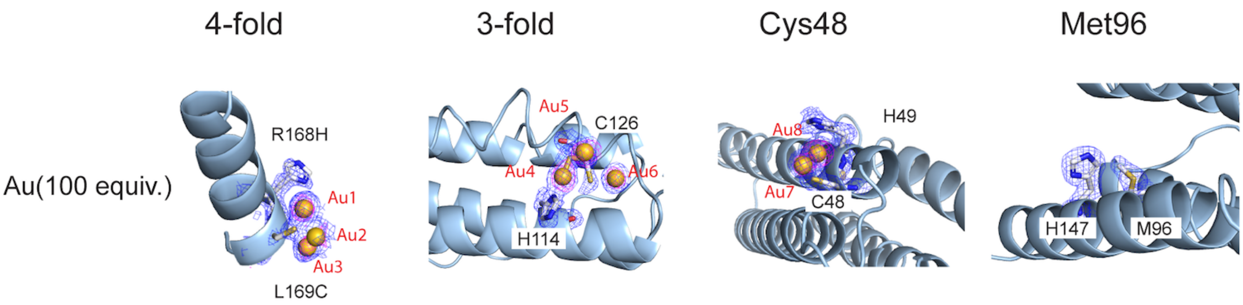

Atom	Au1	Au2	Au3	Au4	Au5	Au6	Au7	Au8
Occu.	0.60	0.30	0.30	0.60	0.70	0.40	0.30	0.90
B.F.(Å^3)	46.27	36.26	37.99	25.90	29.03	30.59	22.97	22.92

Table S6 Bond distances of Au atoms with adjacent amino acids in Au(100 equiv.)-apo-R168H/L169C-rHLFr.

Bond	Bond distance (Å)	Bond	Bond distance (Å)
Au1-S^γ (L169C)	2.59	Au6- S^γ (C126)	2.59
Au2-S^γ (L169C)	2.01	Au7- S^γ (C48)	2.72
Au3- S^γ (L169C)	2.61	Au7- S^γ (C126)	2.34
Au4- N^ε (H114)	2.04	Au8- N^ε (H49)	3.05
Au4- S^γ (C126)	1.87	Au8- N^δ (H49)	3.15
Au5- S^γ (C126)	2.43	Au8- S^γ (C48)	2.34

Table S7 B-Factors (B.F.) and occupancies (Occu.) of Au atoms in Au(200 equiv.)-apo-R168H/L169C-rHLFr

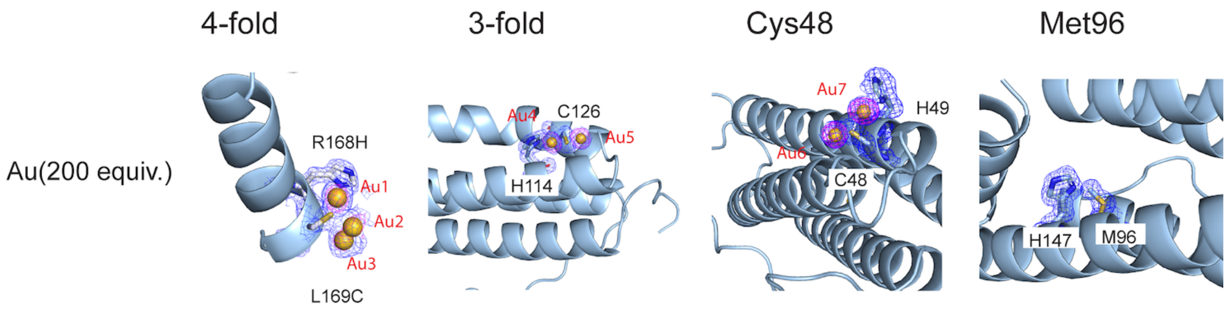

Atom	Au1	Au2	Au3	Au4	Au5	Au6	Au7
Occu.	0.65	0.30	0.30	0.80	0.60	0.70	0.90
B.F.(Å^3)	31.10	21.83	23.76	19.67	14.83	12.23	15.29

Table S8 Bond distances of Au atoms with adjacent amino acids in Au(200 equiv.)-apo-R168H/L169C-rHLFr

Bond	Bond distance (Å)	Bond	Bond distance (Å)
Au1-S^γ (L169C)	2.08	Au5- S^γ (C126)	2.02
Au1- N^ε (R168H)	2.02	Au6- S^γ (C126)	2.09
Au2- S^γ (L169C)	2.10	Au7- S^γ (C48)	2.40
Au3- S^γ (L169C)	2.64	Au7- N^ε (H49)	1.98
Au4- N^ε (H114)	2.06		
Au4- S^γ (C126)	2.40		

Table S9 B-Factors (B.F.) and occupancies (Occu.) of Au atoms in Au(400 equiv.)-apo-R168H/L169C-rHLFr

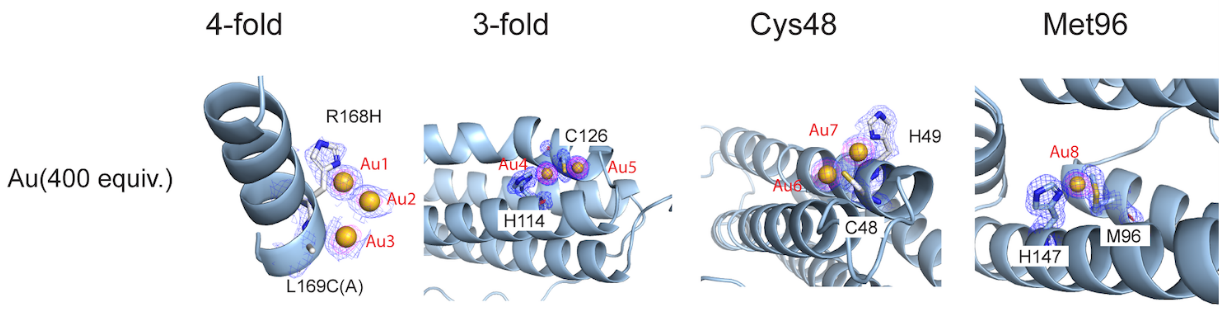

Atom	Au1	Au2	Au3	Au4	Au5	Au6	Au7	Au8
Occu.	0.70	0.50	0.60	0.80	0.80	1.00	1.00	0.43
B.F.(Å^3)	51.83	38.34	42.03	17.36	17.97	17.11	18.55	20.60

Table S10 Bond distances of Au atoms with adjacent amino acids in Au(400 equiv.)-apo-R168H/L169C-rHLFr

Bond	Bond distance (Å)	Bond	Bond distance (Å)
Au3- N$^\varepsilon$ (R168H)	2.48	Au7- N$^\varepsilon$ (H49)	2.00
Au4- N$^\varepsilon$ (H114)	1.99	Au8- S$^\delta$ (M96)	2.51
Au4- S$^\gamma$ (C126)	2.34		
Au5-S$^\gamma$ (C126)	2.08		
Au6- N$^\varepsilon$ (C48)	2.16		
Au7- S$^\gamma$ (C48)	2.35		

Table S11 Representative Au–Au bond distances observed in Au^I clusters in previous reports.

No	Compound	Au–Au distance (Å)	Reference
1	Iodo(tetrahydrothiophene)gold(I)	2.967	[2]
2	$Au_{11}I_3[P(p\text{-}ClC_6H_4)_3]_7$	2.600	[3]
3	$Au_{24}(SCH_2Ph\text{-}^tBu)_{20}$	2.70	[4]
4	$[Au_2(hpp)_2Cl_2]$	2.4752	[5]
5	$CsAu_3S_2$	3.096	[6]
6	$[(PhCOO)_2Au_4(hpp)_4Ag_2(PhCOO)_4]$	2.4473	[7]
7	$[Au_2(PPh_3)_2(\mu\text{-}SCH_2Ph)](NO_3)$	3.077	[8]
8	$[(CF_3)_4Au_2(C_5H_5N)_2]$	2.5062	[9]
9	$[Au_2(2,6\text{-}Me_2Ph\text{-}form)_2(NO3)_2]$	2.486	[10]
10	$[Au_{10}(R\text{-}BINAP)_2(S\text{-}BINAP)_2(\mu_3\text{-}S)4]Cl_2$	2.980-3.248	[11]
11	Au_{10}/Au_{18} μ3-sulfido clusters	2.88–3.16	[12]
12	Octanuclear gold(I) alkynyl-diphosphine clusters	2.94–3.18	[13]
13	$[Au_2Ag_2(R^I/R^{II})4](R^I = 4\text{-}C_6F_4I, R^{II} = 2\text{-}C_6F_4I)]$	3.0212	[14]
14	$[Au(I)_{11}SR_{11}]$	2.908–3.288	[15]

Table S12 Representative Au–S bond distances observed in previous reports.

No	Compound	Au–S distance (Å)	Reference
1	$Na_3[Au(S_2O_3)_2]\cdot 2H20$	2.265, 2.279	[16]
2	$[Et_3PAuSR]$	2.328	[17]
3	$Au(S_2CN(n\text{-}C_4H_9)_2)_2AgBr_2$	2.357, 2.324	[18]
4	$[Au\{(PPh_2)_2C_2B_{10}H_{10}\}\{(SPPh_2)_2CH_2\}]ClO_4\cdot CH_2Cl_2$	2.485, 2.661	[19]
5	$[Fe(C_5Me_5)_2][Au(C_3S_5)_2]$	2.312, 2.322	[20]
6	$(C_6H_5)_3PAuSSCN(C_2H_5)2$	2.338	[21]
7	Iodo(tetrahydrothiophene)gold(I)	2.306, 2.335	[2]

Table S13 Representative Au–N bond distances observed in previous reports.

No	Compound	Au–N distance (Å)	Reference
1	$[Au(bipy^R)(\eta^2\text{-CH2=CHPh})][PF_6]$	2.217, 2.150	[22]
2	$Au(dien)Cl_3$	2.048, 2.010	[23]
3	$[Au(terpy)Cl]Cl_2\cdot 3H_2$	2.029, 1.931, 2.018	[24]
4	$Et_3PAu(1\text{-Methy})$	2.06	[25]
5	$[(EtO)(MeC_6H_5N=)CAu]_3$	2.018, 2.037, 2.045	[26]
6	AuCN	1.82	[27]
7	$[Au(phen)\{(CN)_{0.92}Br_{0.08}\}_2]Br$	2.14, 2.02	[28]

IV. Supplementary references

1. Abe, S. *et al.* Coordination design of cadmium ions at the 4-fold axis channel of the apo-ferritin cage. *Dalt. Trans.* 9759–9764 (2019) doi:10.1039/c9dt00609e.
2. Ahrland, S., Noren, B. & Oskarsson, A. Crystal Structure of Iodo(tetrahydrothiophene)gold(I) at 200 K: A Compound with an Infinite Array of Gold-Gold Bonds. *Inorg. Chem.* **24**, 1330–1333 (1985).
3. Meunier-Piret, J., Van Meerssche, M., Jurkschat, K. & Gielen, M. Crystal and molecular structure of bis(chlorodiphenylstannyl)methane. *J. Organomet. Chem.* **288**, 139–143 (1985).
4. Das, A. *et al.* Crystal structure and electronic properties of a thiolate-protected Au 24 nanocluster. *Nanoscale* **6**, 6458–6462 (2014).
5. Irwin, M. D., Abdou, H. E., Mohamed, A. A. & Fackler, J. P. Synthesis and X-ray structures of silver and gold guanidinate-like complexes. A Au(ii) complex with a 2.47 Å Au-Au distance. *Chem. Commun.* **3**, 2882–2883 (2003).
6. Klepp, K. O. & Weithaler, C. The crystal structures of CsAu3S2, RbAu3Se2 and CsAu3Se2 and their relationship to the CsCu3S2 structure type. *J. Alloys Compd.* **243**, 1–5 (1996).
7. Mohamed, A. A., Abdou, H. E., Mayer, A. & Fackler, J. P. A silver(I)-gold(II) hexanuclear guanidinate-benzoate cluster with short Au-Au bonds. *J. Clust. Sci.* **19**, 551–559 (2008).
8. Wang, S. & Fackler, J. P. Gold Thiolate Complexes with Short Intermolecular Au-Au Distances from Reactions of Organic Disulfides with Gold(I) Complexes. Syntheses and Crystal Structures of [AuI2(PPh3)2(μ-SCH2Ph)](NO3) and AuIII2Cl4(μ-SPh)2. *Inorg.*

Chem. **29**, 4404–4407 (1990).

9. Zopes, D., Hegemann, C., Tyrra, W. & Mathur, S. [(CF3)4Au2(C5H5N)2] – a new alkyl gold(ii) derivative with a very short Au–Au bond. *Chem. Commun.* **48**, 8805–8807 (2012).

10. Melgarejo, D. Y. *et al.* Synthesis and structure of a dinuclear gold(II) complex with terminal fluoride ligands. *Inorg. Chem.* **50**, 4238–4240 (2011).

11. Yao, L. Y., Lee, T. K. M. & Yam, V. W. W. Thermodynamic-Driven Self-Assembly: Heterochiral Self-Sorting and Structural Reconfiguration in Gold(I)-Sulfido Cluster System. *J. Am. Chem. Soc.* **138**, 7260–7263 (2016).

12. Yao, L. Y. & Yam, V. W. W. Photoinduced Isomerization-Driven Structural Transformation between Decanuclear and Octadecanuclear Gold(I) Sulfido Clusters. *J. Am. Chem. Soc.* **137**, 3506–3509 (2015).

13. Koshevoy, I. O. *et al.* Octanuclear gold(i) alkynyl-diphosphine clusters showing thermochromic luminescence. *Chem. Commun.* **47**, 5533–5535 (2011).

14. Lasanta, T., Olmos, M. E., Laguna, A., López-De-Luzuriaga, J. M. & Naumov, P. Making the golden connection: Reversible mechanochemical and vapochemical switching of luminescence from bimetallic gold-silver clusters associated through aurophilic interactions. *J. Am. Chem. Soc.* **133**, 16358–16361 (2011).

15. Chui, S. S. Y., Chen, R. & Che, C. M. A chiral [2]catenane precursor of the antiarthritic gold(I) drug auranofin. *Angew. Chemie - Int. Ed.* **45**, 1621–1624 (2006).

16. Ssoz, N. A. AND MOLECULAR STRUCTURE OF DITHIOSULPHATE AURATE (I), DIHYDRATE , THE T H I O S U L P H A T E anion has been demonstrated to be a quite versatile iigand . It can co-ordinate to transition metals through sulphur , oxygen or both , as has been determined . **35**, 3191–3200 (1973).

17. *X-ray crystal structure of (6-n-propyl-2- thiouracilato) (triethylphosphine) gold (I).* vol. 23 231–234 (1993).

18. Cras, J. A., Noordik, J. H., Beurskens, P. T. & Verhoeven, A. M. Crystal and molecular structure of bis (N , N-di-n-butyldithiocarbamato) gold (H]) dibromoargentate (1). 155–160 (1970).

19. Crespo, O., Gimeno, M. C., Jones, P. G. & Laguna, A. Mixed Four-Coordinate Gold(I) Complexes with Diphosphines or Diphosphine Disulfides as Ligands. *Inorg. Chem.* **33**, 6128–6131 (1994).

20. Matsubayashi, G. etsu & Yokozawa, A. X-ray crystal structure of [Fe(C5Me5)2][Au(C3S5)2] and properties of partially oxidized [Au(C3S5)2] anion complexes. *Inorganica Chim. Acta* **193**, 137–141 (1992).

21. Wijnhoven, J. G., Bosman, W. P. J. H. & Beurskens, P. T. Crystal and molecular structure of triphenylphosphine (N,N-diethyldithiocarbamato)gold(I). *J. Cryst. Mol. Struct.* **2**, 7–15 (1972).

22. Cinellu, M. A., Minghetti, G., Stoccoro, S., Zucca, A. & Manassero, M. Reaction of gold(III) oxo complexes with alkenes. Synthesis of unprecedented gold alkene complexes, [Au(N, N)(alkene)][PF6]. Crystal structure of [Au(bipyip)(η2-CH2≡CHPh)][PF6] (bipyip = 6-isopropyl-2,2′-bipyridine). *Chem. Commun.* **4**, 1618–1619 (2004).

23. Elder, R. C. & Watkins, J. W. Structure of Trichloro(diethylenetriamine)gold(III), Au(dien)Cl3, Determined by Single-Crystal X-ray Diffraction, Raman, and EXAFS Spectroscopies: An EXAFS Caveat. *Inorg. Chem.* **25**, 223–226 (1986).

24. Hollis, L. S. & Lippard, S. J. Aqueous Chemistry of (2,2’,2″ -Terpyridine)gold(III). Preparation and Structures of [Au(terpy)Cl]Cl2.3H2O and the Mixed-Valence Au(I)-

Au(III) Salt [Au(terpy)Cl]2[AuCl2]3[AuCl4]. *J. Am. Chem. Soc.* **105**, 4293–4299 (1983).

25. Tiekink, E. R. T. X-ray crystal structure of (1-methylthyminato)triethylphosphinegold(I). *J. Crystallogr. Spectrosc. Res.* **20**, 371–374 (1990).

26. Tiripicchio, A., Camellini, M. T. & Minghetti, G. The crystal structure of tris-μ-[(ethoxy)(N-p-tolylimino)methyl-N, C]trigold(I), [(EtO)(MeC6H4N)CAu]3. *J. Organomet. Chem.* **171**, 399–406 (1979).

27. Bowmaker, G. A., Kennedy, B. J. & Reid, J. C. Crystal Structures of AuCN and AgCN and Vibrational Spectroscopic Studies of AuCN, AgCN, and CuCN. *Inorg. Chem.* **37**, 3968–3974 (1998).

28. Marangoni, G., Pitteri, B., Bertolasi, V., Ferretti, V. & Gilli, G. p = 105.10(2),. (1987).
