# Supplementary material for: Design of a gold clustering site in an engineered apo-ferritin cage
Source: Commun Chem. 2022 Mar 21;5:39. doi: 10.1038/s42004-022-00651-1 (PMC9814837; doi:10.1038/s42004-022-00651-1)
Supplement: Supplementary file 2 — Description of Additional Supplementary Files [file 42004_2022_651_MOESM2_ESM.pdf]

Description of Additional Supplementary Files

File Name: Supplementary Data 1

Description: The atomic coordinates of the optimized computational models
